# Supplementary material for: Identification of genes and functional coexpression modules closely related to ulcerative colitis by gene datasets analysis
Source: PeerJ. 2019 Nov 13;7:e8061. doi: 10.7717/peerj.8061 (PMC6858811; doi:10.7717/peerj.8061)
Supplement: Supplemental Information 1 [file peerj-07-8061-s001.doc]

**Table S1 Top 100 up-regulated and top 50 down-regulated genes in UC** patients

| **Gene name** | **Symbol** | **Type** | **logFC** | **P value** | **adjPvalue** |
| --- | --- | --- | --- | --- | --- |
| **Matrix metallopeptidase 1** | MMP1 | Up-regulated | 3.270834193 | 9.37E-17 | 2.70E-12 |
| **regenerating islet-derived 1 alpha** | REG1A | Up-regulated | 3.946628081 | 1.92E-16 | 5.52E-12 |
| **deleted in malignant brain tumors 1** | DMBT1 | Up-regulated | 2.40014572 | 4.16E-14 | 1.20E-09 |
| **dual oxidase 2** | DUOX2 | Up-regulated | 2.819323362 | 5.32E-13 | 1.53E-08 |
| **regenerating islet-derived 3 alpha** | REG3A | Up-regulated | 2.78492178 | 6.40E-13 | 1.84E-08 |
| **dual oxidase maturation factor 2** | DUOXA2 | Up-regulated | 2.740412898 | 7.54E-13 | 2.17E-08 |
| **lipocalin 2** | LCN2 | Up-regulated | 3.113171796 | 8.27E-13 | 2.38E-08 |
| **matrix metallopeptidase 10** | MMP10 | Up-regulated | 1.869354719 | 3.71E-12 | 1.07E-07 |
| **matrix metallopeptidase 3** | MMP3 | Up-regulated | 2.848789223 | 5.81E-12 | 1.67E-07 |
| **selectin L** | SELL | Up-regulated | 1.634160933 | 6.28E-12 | 1.81E-07 |
| **S100 calcium binding protein A9** | S100A9 | Up-regulated | 2.023101642 | 8.02E-12 | 2.31E-07 |
| **S100 calcium binding protein A8** | S100A8 | Up-regulated | 2.492628052 | 1.21E-11 | 3.47E-07 |
| **chemokine (C-X-C motif) ligand 13** | CXCL13 | Up-regulated | 1.924603346 | 1.26E-11 | 3.64E-07 |
| **chemokine (C-X-C motif) ligand 8** | CXCL8 | Up-regulated | 2.156022338 | 1.42E-11 | 4.09E-07 |
| **peptidase inhibitor 3** | PI3 | Up-regulated | 2.247296059 | 2.57E-11 | 7.40E-07 |
| **chitinase 3-like 1** | CHI3L1 | Up-regulated | 2.361023713 | 2.94E-11 | 8.47E-07 |
| **regenerating islet-derived 1 beta** | REG1B | Up-regulated | 3.135830728 | 3.81E-11 | 1.10E-06 |
| **chemokine (C-X-C motif) ligand 11** | CXCL11 | Up-regulated | 2.058489893 | 4.85E-11 | 1.40E-06 |
| **matrix metallopeptidase 7** | MMP7 | Up-regulated | 2.136820559 | 1.13E-10 | 3.25E-06 |
| **olfactomedin 4** | OLFM4 | Up-regulated | 2.1427345 | 1.81E-10 | 5.21E-06 |
| **Pim-2 proto-oncogene** | PIM2 | Up-regulated | 1.458912745 | 2.42E-10 | 6.96E-06 |
| **solute carrier family 6 (amino acid transporter), member 14** | SLC6A14 | Up-regulated | 2.682092451 | 2.62E-10 | 7.53E-06 |
| **complement factor B** | CFB | Up-regulated | 1.421827709 | 3.56E-10 | 1.02E-05 |
| **interleukin 1, beta** | IL1B | Up-regulated | 1.811105587 | 5.36E-10 | 1.54E-05 |
| **matrix metallopeptidase 12** | MMP12 | Up-regulated | 2.121320079 | 7.11E-10 | 2.05E-05 |
| **serine peptidase inhibitor, Kazal type 4** | SPINK4 | Up-regulated | 2.020640062 | 7.76E-10 | 2.23E-05 |
| **matrix metallopeptidase 9** | MMP9 | Up-regulated | 1.73023197 | 9.75E-10 | 2.81E-05 |
| **stanniocalcin 1** | STC1 | Up-regulated | 1.31747263 | 1.35E-09 | 3.87E-05 |
| **interleukin 1 receptor antagonist** | IL1RN | Up-regulated | 1.417897515 | 1.38E-09 | 3.99E-05 |
| **vanin 1** | VNN1 | Up-regulated | 2.00196378 | 1.55E-09 | 4.46E-05 |
| **chemokine (C-C motif) ligand 19** | CCL19 | Up-regulated | 1.365503142 | 1.57E-09 | 4.52E-05 |
| **indoleamine 2,3-dioxygenase 1** | IDO1 | Up-regulated | 2.016283413 | 1.81E-09 | 5.20E-05 |
| **chemokine (C-C motif) ligand 20** | CCL20 | Up-regulated | 1.411984031 | 2.08E-09 | 5.98E-05 |
| **chemokine (C-X-C motif) ligand 10** | CXCL10 | Up-regulated | 1.634205444 | 2.17E-09 | 6.26E-05 |
| **TIMP metallopeptidase inhibitor 1** | TIMP1 | Up-regulated | 1.695099837 | 2.28E-09 | 6.55E-05 |
| **CD27 molecule** | CD27 | Up-regulated | 1.228546364 | 2.85E-09 | 8.22E-05 |
| **plasminogen activator, urokinase** | PLAU | Up-regulated | 1.446910386 | 2.95E-09 | 8.50E-05 |
| **colony stimulating factor 3 receptor** | CSF3R | Up-regulated | 1.06612297 | 3.22E-09 | 9.28E-05 |
| **TNFAIP3 interacting protein 3** | TNIP3 | Up-regulated | 2.000789092 | 3.63E-09 | 0.000104397 |
| **trefoil factor 1** | TFF1 | Up-regulated | 1.267444182 | 3.71E-09 | 0.000106887 |
| **adrenomedullin** | ADM | Up-regulated | 1.137728232 | 3.98E-09 | 0.000114555 |
| **complement component 2** | C2 | Up-regulated | 1.209998962 | 4.02E-09 | 0.000115875 |
| **S100 calcium binding protein P** | S100P | Up-regulated | 1.465302875 | 4.87E-09 | 0.000140236 |
| **insulin-like growth factor binding protein 5** | IGFBP5 | Up-regulated | 1.43654411 | 5.20E-09 | 0.000149751 |
| **lymphocyte transmembrane adaptor 1** | LAX1 | Up-regulated | 1.273100578 | 7.03E-09 | 0.000202302 |
| **complement factor I** | CFI | Up-regulated | 1.489416817 | 7.27E-09 | 0.000209242 |
| **chemokine (C-X-C motif) ligand 1** | CXCL1 | Up-regulated | 2.438798098 | 8.84E-09 | 0.000254699 |
| **aquaporin 9** | AQP9 | Up-regulated | 1.898673205 | 9.33E-09 | 0.000268555 |
| **serpin peptidase inhibitor, clade B (ovalbumin), member 5** | SERPINB5 | Up-regulated | 1.80094097 | 1.20E-08 | 0.000346419 |
| **tryptophanyl-tRNA synthetase** | WARS | Up-regulated | 1.219573693 | 1.32E-08 | 0.000379428 |
| **defensin, alpha 5** | DEFA5 | Up-regulated | 2.21809999 | 1.67E-08 | 0.000481851 |
| **kynureninase** | KYNU | Up-regulated | 1.585797654 | 1.74E-08 | 0.000500214 |
| **platelet/endothelial cell adhesion molecule 1** | PECAM1 | Up-regulated | 1.11747822 | 2.17E-08 | 0.000624221 |
| **cathepsin K** | CTSK | Up-regulated | 1.091314752 | 2.26E-08 | 0.000649555 |
| **nitric oxide synthase 2** | NOS2 | Up-regulated | 1.697376496 | 2.49E-08 | 0.000717793 |
| **thrombospondin 2** | THBS2 | Up-regulated | 1.128897872 | 2.61E-08 | 0.000750671 |
| **serglycin** | SRGN | Up-regulated | 1.208877261 | 2.73E-08 | 0.000786812 |
| **chemokine (C-C motif) receptor 7** | CCR7 | Up-regulated | 1.075774761 | 2.84E-08 | 0.000817982 |
| **chemokine (C-C motif) ligand 11** | CCL11 | Up-regulated | 1.287009717 | 4.33E-08 | 0.001246523 |
| **guanine nucleotide binding protein , alpha 15** | GNA15 | Up-regulated | 1.082809744 | 4.81E-08 | 0.001385866 |
| **pleckstrin** | PLEK | Up-regulated | 1.258672681 | 5.12E-08 | 0.001474885 |
| **selectin P** | SELP | Up-regulated | 1.218330419 | 6.14E-08 | 0.001767772 |
| **retinoic acid receptor responder 3** | RARRES3 | Up-regulated | 1.015624074 | 6.28E-08 | 0.001807426 |
| **neutrophil cytosolic factor 2** | NCF2 | Up-regulated | 1.212532999 | 6.36E-08 | 0.001831428 |
| **guanylate binding protein 5** | GBP5 | Up-regulated | 1.206824672 | 6.51E-08 | 0.001874453 |
| **myeloid cell nuclear differentiation antigen** | MNDA | Up-regulated | 1.205594153 | 7.34E-08 | 0.002112692 |
| **chemokine (C-X-C motif) ligand 6** | CXCL6 | Up-regulated | 1.468793064 | 7.97E-08 | 0.002294476 |
| **serpin peptidase inhibitor, clade A, member 3** | SERPINA3 | Up-regulated | 1.401627432 | 8.93E-08 | 0.002572876 |
| **annexin A1** | ANXA1 | Up-regulated | 1.394107838 | 9.26E-08 | 0.002666277 |
| **complement component 4 binding protein, beta** | C4BPB | Up-regulated | 1.484189976 | 9.76E-08 | 0.002811112 |
| **angiopoietin-like 2** | ANGPTL2 | Up-regulated | 1.073116811 | 9.89E-08 | 0.002846778 |
| **chemokine (C-C motif) ligand 18** | CCL18 | Up-regulated | 1.153085817 | 1.09E-07 | 0.003150887 |
| **cell migration inducing protein** | CEMIP | Up-regulated | 1.155686976 | 1.16E-07 | 0.003336761 |
| **selectin E** | SELE | Up-regulated | 1.230323603 | 1.26E-07 | 0.003625615 |
| **suppressor of cytokine signaling 3** | SOCS3 | Up-regulated | 1.109330005 | 1.32E-07 | 0.003793618 |
| **complement component 4 binding protein, alpha** | C4BPA | Up-regulated | 1.347765412 | 1.32E-07 | 0.003807681 |
| **tripartite motif containing 22** | TRIM22 | Up-regulated | 1.15223417 | 1.36E-07 | 0.003925024 |
| **chemokine (C-X-C motif) receptor 4** | CXCR4 | Up-regulated | 1.123549528 | 1.39E-07 | 0.003996511 |
| **BCL2-related protein A1** | BCL2A1 | Up-regulated | 1.35148132 | 1.41E-07 | 0.004060986 |
| **interferon induced transmembrane protein 2** | IFITM2 | Up-regulated | 1.020120284 | 1.55E-07 | 0.004464352 |
| **chemokine (C-X-C motif) ligand 2** | CXCL2 | Up-regulated | 1.386884268 | 1.83E-07 | 0.005280924 |
| **PDZK1 interacting protein 1** | PDZK1IP1 | Up-regulated | 1.471478472 | 1.97E-07 | 0.005686954 |
| **regenerating islet-derived family, member 4** | REG4 | Up-regulated | 1.444811101 | 2.03E-07 | 0.005854743 |
| **interferon induced transmembrane protein 3** | IFITM3 | Up-regulated | 1.096861207 | 2.52E-07 | 0.007244173 |
| **tenascin C** | TNC | Up-regulated | 1.444793844 | 2.72E-07 | 0.007832695 |
| **transcobalamin I** | TCN1 | Up-regulated | 1.80423892 | 2.77E-07 | 0.007976853 |
| **caspase 5, apoptosis-related cysteine peptidase** | CASP5 | Up-regulated | 1.024509081 | 2.81E-07 | 0.00807841 |
| **lysophosphatidylcholine acyltransferase 1** | LPCAT1 | Up-regulated | 1.300223813 | 3.50E-07 | 0.010088157 |
| **stomatin** | STOM | Up-regulated | 1.03948761 | 3.64E-07 | 0.010478371 |
| **biglycan** | BGN | Up-regulated | 1.305807077 | 3.66E-07 | 0.010535606 |
| **ubiquitin D** | UBD | Up-regulated | 1.363876792 | 3.67E-07 | 0.010557763 |
| **cell division cycle 25B** | CDC25B | Up-regulated | 1.010226866 | 3.85E-07 | 0.01108474 |
| **oncostatin M receptor** | OSMR | Up-regulated | 1.191367376 | 4.07E-07 | 0.011709664 |
| **GTP binding protein overexpressed in skeletal muscle** | GEM | Up-regulated | 1.130496239 | 4.63E-07 | 0.013336772 |
| **S100 calcium binding protein A12** | S100A12 | Up-regulated | 1.751153684 | 4.74E-07 | 0.013642189 |
| **aldolase B, fructose-bisphosphate** | ALDOB | Up-regulated | 1.202637779 | 4.93E-07 | 0.014185895 |
| **secreted phosphoprotein 1** | SPP1 | Up-regulated | 1.216505486 | 4.98E-07 | 0.014352138 |
| **baculoviral IAP repeat containing 3** | BIRC3 | Up-regulated | 1.106298987 | 5.16E-07 | 0.01485963 |
| **G0/G1 switch 2** | G0S2 | Up-regulated | 1.102960145 | 5.16E-07 | 0.01485963 |
| **vanin 2** | VNN2 | Up-regulated | 1.111728729 | 6.11E-07 | 0.017601498 |
| **aquaporin 8** | AQP8 | Down-regulated | -3.711269886 | 7.89E-19 | 2.27E-14 |
| **claudin 8** | CLDN8 | Down-regulated | -3.502675426 | 6.28E-17 | 1.81E-12 |
| **ATP-binding cassette, sub-family G, member 2** | ABCG2 | Down-regulated | -2.290628662 | 2.38E-13 | 6.87E-09 |
| **3-hydroxy-3-methylglutaryl-CoA synthase 2** | HMGCS2 | Down-regulated | -2.623853201 | 3.16E-13 | 9.11E-09 |
| **phosphoenolpyruvate carboxykinase 1** | PCK1 | Down-regulated | -2.797323761 | 3.63E-13 | 1.05E-08 |
| **guanylate cyclase activator 2B** | GUCA2B | Down-regulated | -2.191758899 | 6.40E-13 | 1.84E-08 |
| **guanylate cyclase activator 2A** | GUCA2A | Down-regulated | -2.167271709 | 4.74E-12 | 1.36E-07 |
| **PH domain and leucine rich repeat protein phosphatase 2** | PHLPP2 | Down-regulated | -1.474983187 | 5.47E-11 | 1.58E-06 |
| **flavin containing monooxygenase 5** | FMO5 | Down-regulated | -1.307346471 | 6.42E-11 | 1.85E-06 |
| **RUN domain containing 3B** | RUNDC3B | Down-regulated | -1.279924689 | 1.08E-10 | 3.11E-06 |
| **solute carrier family 26 (anion exchanger), member 2** | SLC26A2 | Down-regulated | -2.671450197 | 2.62E-10 | 7.53E-06 |
| **hepatitis A virus cellular receptor 1** | HAVCR1 | Down-regulated | -1.279287083 | 2.89E-10 | 8.31E-06 |
| **calcineurin-like EF-hand protein 2** | CHP2 | Down-regulated | -1.849386744 | 3.49E-10 | 1.01E-05 |
| **exophilin 5** | EXPH5 | Down-regulated | -1.490486931 | 3.57E-10 | 1.03E-05 |
| **meprin A, alpha** | MEP1A | Down-regulated | -1.421034817 | 4.70E-10 | 1.35E-05 |
| **tetraspanin 7** | TSPAN7 | Down-regulated | -1.285129262 | 5.74E-10 | 1.65E-05 |
| **selenium binding protein 1** | SELENBP1 | Down-regulated | -1.376857162 | 6.48E-10 | 1.87E-05 |
| **metallothionein 1F** | MT1F | Down-regulated | -1.201890126 | 8.71E-10 | 2.51E-05 |
| **hydroxysteroid (17-beta) dehydrogenase 2** | HSD17B2 | Down-regulated | -1.182485296 | 2.46E-09 | 7.08E-05 |
| **cadherin-related family member 1** | CDHR1 | Down-regulated | -1.192692688 | 3.08E-09 | 8.87E-05 |
| **creatine kinase, brain** | CKB | Down-regulated | -1.427558604 | 3.21E-09 | 9.24E-05 |
| **ectonucleoside triphosphate diphosphohydrolase 5** | ENTPD5 | Down-regulated | -1.296902639 | 3.34E-09 | 9.63E-05 |
| **alcohol dehydrogenase 1C** | ADH1C | Down-regulated | -1.702725701 | 4.60E-09 | 0.000132377 |
| **metallothionein 1H** | MT1H | Down-regulated | -1.268953509 | 4.75E-09 | 0.000136817 |
| **vasoactive intestinal peptide receptor 1** | VIPR1 | Down-regulated | -1.175874624 | 5.12E-09 | 0.000147329 |
| **carbonic anhydrase I** | CA1 | Down-regulated | -1.514425895 | 1.42E-08 | 0.000407627 |
| **calpain 13** | CAPN13 | Down-regulated | -1.213032936 | 1.69E-08 | 0.000486845 |
| **ATP-binding cassette, sub-family B, member 1** | ABCB1 | Down-regulated | -1.481876816 | 2.09E-08 | 0.00060166 |
| **SATB homeobox 2** | SATB2 | Down-regulated | -1.25110032 | 2.49E-08 | 0.000717793 |
| **transmembrane and immunoglobulin domain containing 1** | TMIGD1 | Down-regulated | -1.528896328 | 2.89E-08 | 0.000832465 |
| **peroxisome proliferator-activated receptor gamma** | PPARG | Down-regulated | -1.012337258 | 3.55E-08 | 0.00102247 |
| **cell wall biogenesis 43 C-terminal homolog** | CWH43 | Down-regulated | -1.242544282 | 4.12E-08 | 0.001185102 |
| **monoamine oxidase A** | MAOA | Down-regulated | -1.166444956 | 5.23E-08 | 0.001505489 |
| **carbonic anhydrase VII** | CA7 | Down-regulated | -1.232163945 | 5.38E-08 | 0.001549035 |
| **apolipoprotein B mRNA editing enzyme, catalytic polypeptide-like 3B** | APOBEC3B | Down-regulated | -1.251063886 | 5.64E-08 | 0.0016254 |
| **N-acylethanolamine acid amidase** | NAAA | Down-regulated | -1.03540306 | 5.79E-08 | 0.001668639 |
| **serum/glucocorticoid regulated kinase 2** | SGK2 | Down-regulated | -1.051304073 | 1.12E-07 | 0.003226187 |
| **prostaglandin D2 receptor** | PTGDR | Down-regulated | -1.112614981 | 1.16E-07 | 0.003336761 |
| **bone morphogenetic protein 3** | BMP3 | Down-regulated | -1.457147133 | 1.35E-07 | 0.003880824 |
| **bestrophin 4** | BEST4 | Down-regulated | -1.191884929 | 1.50E-07 | 0.00432671 |
| **sclerostin domain containing 1** | SOSTDC1 | Down-regulated | -1.005259243 | 1.65E-07 | 0.004749451 |
| **CD177 molecule** | CD177 | Down-regulated | -1.068213689 | 1.90E-07 | 0.005481438 |
| **metallothionein 1G** | MT1G | Down-regulated | -1.351120455 | 2.15E-07 | 0.006204551 |
| **peptidyl arginine deiminase, type II** | PADI2 | Down-regulated | -1.424087948 | 2.61E-07 | 0.007517159 |
| **contactin 3** | CNTN3 | Down-regulated | -1.233316083 | 2.77E-07 | 0.007976853 |
| **HEPACAM family member 2** | HEPACAM2 | Down-regulated | -1.129441637 | 2.77E-07 | 0.007976853 |
| **transient receptor potential cation channel, subfamily M, member 6** | TRPM6 | Down-regulated | -1.229148333 | 4.51E-07 | 0.012982878 |
| **dehydrogenase/reductase (SDR family) member 11** | DHRS11 | Down-regulated | -1.169559757 | 5.65E-07 | 0.016278679 |
| **ciliary neurotrophic factor receptor** | CNTFR | Down-regulated | -1.160110866 | 6.00E-07 | 0.017274802 |
| **metallothionein 1M** | MT1M | Down-regulated | -1.50138124 | 6.32E-07 | 0.018192391 |
