# Supplementary material for: Identification of genes and functional coexpression modules closely related to ulcerative colitis by gene datasets analysis
Source: PeerJ. 2019 Nov 13;7:e8061. doi: 10.7717/peerj.8061 (PMC6858811; doi:10.7717/peerj.8061)
Supplement: Supplemental Information 2 [file peerj-07-8061-s002.doc]

**Table S2 Seven main functional co-expression modules and related genes involved in** UC

| **Co-Expression Modules** | **Number of Genes(number of top150)** | **Genes in modules** |
| --- | --- | --- |
| Blue module | 1718(38/150) | A2M AAED1 AARS AASS ABCC1 ABCC3 ABHD11 ABHD14A ABL2 ABLIM3 ABO ABR ACD ACE2 ACKR1 ACKR3 ACO2 ACOT11 ACP5 ACPP ACRC ACSL4 ACTN1 ADA ADAM12 ADAM19 ADAM22 ADAMDEC1 ADAMTS1 ADAMTS2 ADAP1 ADCY3 ADCY4 ADCY7 ADGRA2 ADGRF5 ADGRG5 ADGRL2 ADGRL4 ADH1A ADH1B ADPGK ADPRH ADPRHL1 AEBP1 AEN AFAP1L1 AGFG2 AGR2 AGT AGTRAP AHNAK2 AHR AIM2 AK1 AK7 AKAP10 AKAP5 AKIRIN2 AKR1B1 AKR7A3 ALDH1L2 ALDH3B1 ALOX5 ALPI ALPK2 AMOTL2 AMPD1 AMPD2 AMPD3 AMT ANGEL2 ANGPT2 **ANGPTL2** ANKEF1 ANKRD22 ANKRD44 ANKRD46 ANKRD49 ANO6 ANO7 ANTXR1 ANXA2P1 ANXA4 ANXA5 ANXA6 AOX1 APBA1 APBA2 APCDD1 APLNR APOBEC3F APOBEC3G APOC1 APOE AQP1 ARFGAP2 ARHGAP1 ARHGAP24 ARHGAP25 ARHGAP27 ARHGAP28 ARHGAP29 ARHGAP30 ARHGAP32 ARHGAP35 ARHGAP4 ARHGAP9 ARHGDIA ARHGDIB ARHGEF12 ARHGEF3 ARHGEF6 ARID3A ARID5A ARID5B ARL4C ARMCX6 ARNTL2 ARPC1B ARSE ASAP1 ASMTL ASPH ATF4 ATM ATOX1 ATP10D ATP13A4 ATP8B2 ATRX AVL9 AVPR1A B4GALNT2 BACE1 BAG1 BAG2 BAK1 BAMBI BANK1 BASP1 BATF BCAT1 BCAT2 BCHE BCKDK BCL11A BCL2 BCL2L10 BCL2L14 BDKRB2 BEND7 BEX4 **BGN** BICC1 BICD1 BIN2 BLK BLM BLNK BLVRA **BMP3** BMP6 BNIP3 BST2 BTG2 BTK BTLA BTN2A2 C10orf10 C11orf80 C11orf96 C16orf45 C1orf162 C1R C1S C20orf194 **C2** CD2 C2orf42 C2orf72 C3 C3orf52 C4B_2 C9orf152 CA12 CADM1 CALCRL CALD1 CALHM2 CALU CAP2 CAPN5 CARD6 CARD8 CASP4 CASZ1 CAV1 CAV2 CBX4 CBY1 CC2D1A CCDC13 CCDC3 CCDC69 CCDC80 CCDC88A **CCL11** **CCL18** **CCL19** CCL2 CCL21 CCL22 CCNI CCR10 CCR2 CCR5 CCR6 **CCR7** CD109 CD163 **CD177** CD180 CD19 CD1C CD2 CD200 CD22 CD226 CD247 CD248 **CD27** CD276 CD28 CD2AP CD34 CD37 CD38 CD3D CD3E CD3G CD40 CD40LG CD44 CD48 CD5 CD52 CD53 CD59 CD69 CD7 CD72 CD79A CD79B CD80 CD81 CD82 CD83 CD84 CD86 CD8A CD9 CD93 CDC42EP3 CDH11 CDH13 CDH5 CDIPT CDK13 CDK14 CDK18 CDK5R1 CDKL1 CDKN2C CDS1 CDYL2 CECR1 **CEMIP** CEP128 CEP135 CEP19 CEP350 CEP85L CERK CETP CFH CFL2 CH25H CHAD CHD3 CHD4 CHDH **CHI3L1** CHI3L2 CHN1 CHPF2 CHRDL1 CHRDL2 CHRM3 CHST11 CHST15 CHST2 CHST3 CHST7 CHSY1 CHSY3 CKAP2 CLDN12 CLDN3 CLDN4 CLDN7 CLEC14A CLEC2B CLECL1 CLIC2 CLIC4 CLIP3 CLMP CLNS1A CLPTM1L CLU CLUH CMBL CMKLR1 CMTM7 CNFN CNPPD1 CNPY2 **CNTFR** CNTNAP2 CNTRL COL12A1 COL14A1 COL15A1 COL18A1 COL1A1 COL1A2 COL3A1 COL4A1 COL4A2 COL5A2 COL6A2 COL6A3 COL7A1 COL8A1 COQ10A CORO1A COTL1 COX6A1 COX7A1 CPEB4 CPNE5 CPVL CPXM1 CR1 CR1L CR2 CRACR2A CRAT CRB3 CRBN CREB3L1 CREB3L2 CREBL2 CREBZF CREM CRIP2 CRISPLD2 CRLF3 CRTAM CRTC3 CSF1R CSF2RA CSGALNACT1 CSGALNACT2 CSK CSRP2 CST7 CSTA CTGF CTHRC1 CTLA4 CTSB CTSC CTSE CTSH **CTSK** CTSL CX3CL1 CXCL12 **CXCL13** **CXCL6** CXCL9 CXCR3 **CXCR4** CXCR5 CXCR6 CXorf21 CYB561 CYBA CYFIP2 CYGB CYP26B1 CYP3A4 CYR61 CYSLTR1 CYTH1 CYTH4 CYTIP CYYR1 DAB2IP DAPP1 DCHS1 DCLK1 DCN DDAH1 DDB2 DDC DDHD2 DDIT4 DDR2 DECR2 DEF6 DEF8 DEGS1 DENND1C DENND3 DENND4B DENND5A DERL2 DERL3 DIO2 DIP2C DKK3 DLC1 DLL1 DMD DMTN DNAJB9 DNAJC1 DNASE2 DNM1 DOCK11 DOCK2 DOCK8 DOK2 DOK3 DOK4 DOPEY2 DOT1L DPP10-AS1 DPT DPY19L3 DPYSL2 DPYSL3 DQX1 DRAM1 DUOX1 DUOXA1 DUSP14 DUSP16 DUSP2 DUSP22 DUSP4 DUSP5 E2F5 EAF2 EBF1 EBI3 ECM1 ECSCR EDNRA EDNRB EEF1D EFEMP2 EFNA2 EGFL6 EGFR EGR2 EGR3 EHD2 EHD3 EID2B EIF4B ELK3 ELL ELL2 ELMO1 ELMO2 ELOVL2 ELOVL5 EMB EMCN EMILIN1 EML1 EMP3 ENG ENO2 ENPP2 ENTPD1 ENTPD7 EOGT EOMES EPB41L1 EPHX4 EPS8L1 EPS8L2 EPS8L3 ERCC3 ERG ERI2 ERLEC1 ERN1 ERO1B ESAM ESPN ESYT1 ETS1 EVI2A EVI2B EVL EXD2 EXOSC7 EXT2 F2R F2RL2 F3 FAAH FABP2 FADS3 FAM101B FAM120A FAM127A FAM129A FAM20A FAM214B FAM229B FAM30A FAM46C FAM49A FAM65B FAM89B FAP FAT4 FBLN1 FBLN2 FBLN5 FBN1 FBXL7 FBXO9 FCGR2B FCHSD2 FCMR FCN3 FCRL2 FCRL3 FCRL4 FCRL5 FCRLA FDCSP FER1L4 FERMT2 FERMT3 FEZ1 FGD5 FGF2 FGF7 FGFR1 FGFR2 FHL5 FIBIN FICD FILIP1L FJX1 FKBP11 FKBP2 FKBP4 FLI1 FLNA FLNC FLT1 FMNL1 FMNL3 FMO1 FMO3 FN1 FNDC1 FOXC1 FOXF1 FOXN3 FOXQ1 FPR3 FRMD6 FRY FSCN1 FSTL1 FSTL3 FTSJ1 FUT11 FUT8 FXYD5 FYB FYN FZD2 FZD4 FZD7 G6PC GAB2 GAB3 GALE GALNT15 GALNT18 GAS1 GAS7 GATA6 **GEM** GFPT2 GGT5 GIMAP4 GIMAP8 GIPC1 GJA1 GJA4 GJA5 GJB1 GLA GLCCI1 GLG1 GLIPR1 GLIS3 GLT8D1 GLT8D2 GLTSCR1L GM2A GMDS GMFG GNAI1 GNAI2 GNAS GNB5 GNG11 GNPNAT1 GNS GOLGA8A GOLIM4 GOLT1A GOLT1B GP1BA GPC4 GPC6 GPR132 GPR137B GPR155 GPR171 GPR176 GPR18 GPR180 GPR183 GPR19 GPR39 GPR4 GPR68 GPSM3 GPT GPX1 GPX2 GPX7 GPX8 GRAMD1A GRAMD1B GRAP GRB10 GRB7 GREM1 GRHL1 GRIN2C GRK5 GRK6 GRTP1 GSAP GSTO2 GTF3A GTSF1 GUCY1A2 GUCY1A3 GUCY1B3 GYG1 GYG2 GYPC GZF1 GZMH GZMK GZMM H1FX HABP4 HAGLR HAPLN3 HCLS1 HDAC3 HECW2 HEG1 HERPUD1 HGD HGF HHEX HIC1 HIST1H2AM HIVEP2 HK2 HLA-DOB HLF HLX HMGA2 HNF1B HNF4A HOMER1 HOOK3 HOXB2 HOXD1 HR HRCT1 HRH2 HS3ST3A1 HS3ST3B1 HSD11B1 HSH2D HSPA12B HSPA13 HSPG2 HTR4 HTRA1 HUNK HVCN1 HYAL2 ICAM2 ICAM3 ICAM4 ICOS ID1 IDH1 IFFO1 IFI16 IFI30 IFNAR2 IGDCC4 IGF2BP2 IGFBP3 **IGFBP5** IGFBP7 IGFLR1 IGHD IGHM IGHV5-78 IGK IGKC IGLC1 IGLJ3 IGLV6-57 IGSF3 IKBIP IKZF1 IKZF3 IL10RA IL12RB1 IL13RA2 IL16 IL18 IL18R1 IL1R1 IL21R IL24 IL27RA IL2RA IL2RB IL33 IL3RA IL4I1 IL6 IL6R IL7R IMPDH1 INHBA INPP4A INPP5D INTS6L IQCG IQSEC1 IQSEC2 IRAK3 IRF4 IRF8 IRS2 ISLR ITGA5 ITGA8 ITGAL ITGAM ITGAV ITGAX ITGB2 ITGB4 ITGB7 ITK ITPKA ITPR1 ITPRIP JADE1 JAG1 JAG2 JAK1 JAK3 JAM2 JAM3 JAZF1 JPH1 KANK3 KAT6B KATNAL1 KBTBD8 KCNA3 KCNJ8 KCNN3 KDELC1 KDM1B KDR KIAA0040 KIAA0355 KIAA0430 KIAA0922 KIAA0930 KIAA1024 KIAA1161 KIAA1462 KIAA2013 KIF12 KIF21B KIRREL KLC1 KLC2 KLF7 KLHL21 KLHL24 KLHL29 KLHL5 KLHL6 KLK10 KLK3 KLK6 KLRG1 KMO KNTC1 KRT18 KRT19 KRT6A LAD1 LAG3 LAIR1 LAMA4 LAMA5 LAMB2 LAMC1 LAMP3 LAPTM5 LAT LAT2 LATS2 **LAX1** LAYN LBH LCA5 LCK LCP1 LDB2 LDLR LDLRAD3 LEF1 LETM1 LGALS1 LGALS2 LGR4 LHFP LILRB4 LIN7A LINC01123 LMCD1 LMO4 LOC283177 LOC339803 LOC730101 LONRF3 LOX LOXL1 LOXL2 LPAR1 LPCAT3 LPIN1 LPL LPXN LRMP LRP5 LRRC28 LRRC32 LRRC70 LRRK2 LSAMP LSP1 LTA LTB LTBP2 LTF LUM LY6E LY86 LY9 LY96 LYST LYVE1 MACF1 MACROD2 MAD2L2 MAFB MAGED2 MALL MALT1 MAP1B MAP3K13 MAP3K14 MAP3K3 MAP4K1 MAP4K4 MAP7D1 MAP9 MAPKAPK2 MASP1 MB21D1 MBD4 MBOAT2 MCAM MCC MDFIC MDM2 MECOM MED28 MEDAG MEI1 MEOX1 MEOX2 MESDC1 METTL3 MFAP2 MFAP4 MFN2 MFNG MFSD14A MFSD2A MGAT1 MGP MGST1 MGST3 MICB MID1IP1 MID2 MILR1 MIR155 MIR15A MIR8071-2 MLLT11 MMD MME **MMP1** **MMP10** **MMP12** MMP13 MMP14 MMP19 MMP2 **MMP3** **MMP7** **MMP9** MMRN1 MMRN2 MNX1 MOAP1 MPDZ MPG MPV17 MPV17L MPZL2 MR1 MRC2 MRM1 MRPL41 MS4A1 MSC MSL3 MSN MST1R MT3 MTCL1 MTDH MTMR7 MUC1 MUC4 MUC5B MXRA5 MXRA7 MXRA8 MYCBP2 MYCT1 MYD88 MYH10 MYL5 MYL9 MYLK MYO10 MYO1E MYO1G MYO5A MYO5B MYO5C MZB1 N4BP2L1 NAIP NAP1L1 NAP1L2 NAP1L4 NAT8B NCF1 NCF1C NCF4 NCKAP1L NDN NDRG2 NDST2 NELL2 NES NEU1 NEU4 NEURL1 NEXN NFATC1 NFKB2 NFKBIE NGEF NID1 NID2 NKG7 NKTR NLRC3 NME4 NNMT NOD2 NOS1AP NOTCH3 NOX4 NQO2 NR2F1 NRCAM NRDC NREP NRG1 NRN1 NRP1 NRP2 NRROS NRXN3 NUAK1 NUP210 NXN NXPE3 NXT1 OAZ2 ODF3B OGFRL1 OLFM1 OLFML2B ORAI2 ORAI3 OSBPL3 OSBPL8 **OSMR** OSR2 OTOP2 P2RX5 P2RX7 P2RY10 P2RY14 P2RY6 P2RY8 P3H1 PACS1 PAK4 PALM2-AKAP2 PALMD PAM PANX1 PAPLN PAPPA PAPSS1 PAPSS2 PARD6B PARP1 PARP8 PARVB PASK PAX5 PBX3 PCBP3 PCDH12 PCDH17 PCDH7 PCED1B PCLO PCMTD2 PCNX1 PCOLCE PDE10A PDE1C PDE6A PDE9A PDGFRB PDLIM4 PDPN PEA15 PEAR1 **PECAM1** PGF PHACTR1 PHF21A PHGDH PHLDA1 PHTF1 PI15 PIAS3 PIK3AP1 PIK3CD PIK3CG PIK3R3 **PIM2** PIP5K1C PIWIL2 PKD2 PKHD1L1 PKIA PKIG PKLR PKN1 PLA1A PLA2G7 **PLAU** PLBD2 PLCB1 PLCD1 PLCD3 PLCG2 PLEK2 PLEKHG3 PLEKHO1 PLEKHO2 PLK2 PLN PLOD1 PLS3 PLTP PLVAP PLXDC1 PLXDC2 PLXNA1 PLXNB1 PLXNC1 PLXND1 PMEPA1 PMM2 PMP22 PNISR PNLIPRP1 PNOC PODXL PORCN POU2AF1 POU2F2 PPARGC1B PPBP PPOX PPP1R14A PPP1R16B PPP1R9A PPT1 PRAF2 PRDM1 PRDX4 PRELP PREX1 PRKCB PRKCDBP PRKCH PRKD1 PRKD2 PRKD3 PRKG2 PRLR PRNP PROCR PROM2 PROS1 PRR16 PRRX1 PRSS23 PSAT1 PSTPIP1 PTEN PTGDS PTGES PTGFR PTGR1 PTGS1 PTHLH PTP4A3 PTPN14 PTPN3 PTPN6 PTPN7 PTPRB PTPRC PTPRCAP PTPRM PTRF PXDC1 PXDN PYCR1 QKI QPCT RAB13 RAB29 RAB30 RAB31 RAB33A RAB40C RAB8B RABAC1 RABGAP1 RAC2 RALGPS1 RAMP3 RAP1A RAPGEF4 RARRES1 RARRES2 RASA3 RASAL3 RASGRF2 RASGRP1 RASGRP2 RASGRP3 RASIP1 RASSF1 RASSF2 RASSF5 RASSF8 RBM38 RBM6 RBMS1 RBP2 RBPMS RCAN2 RCOR3 RCSD1 RDX RECQL REL RELT RFTN1 RFX5 RGCC RGL2 RGS1 RGS16 RGS19 RGS4 RGS5 RHBDD1 RHBDF2 RHOBTB1 RHOBTB2 RHOH RHOJ RHOQ RILPL2 RIMKLA RIT1 RNF103 RNF38 RNF44 ROBO1 ROBO4 ROR2 RORA RORC RPL22L1 RPN2 RPS6KA2 RRAD RRBP1 RRM2B RSPH1 RSPO3 RTKN RUFY3 RUNX3 S100A16 S100A2 S100A3 S100A4 S100B S1PR1 S1PR4 SACS SAMHD1 SASH1 SASH3 SATB1 SCAI SCN9A SCNN1A SCPEP1 SCRN1 SDC2 SDF2L1 SDHB SDK1 SEC11C SEC14L1 SEC16A SEC22C SEC31A SEC61B SEL1L SEL1L3 **SELE** SELK **SELL** SELM **SELP** SELPLG SEMA4A SEMA4D SEMA4G SEPT1 SEPT6 SEPT9 SERINC2 SERPINA1 **SERPINA3** SERPINB8 SERPINB9 SERPINE1 SERPINE2 SERPINF1 SERPING1 SERPINH1 SERPINI1 SESTD1 SFMBT2 SFRP2 SFXN3 SGIP1 SH2B2 SH2D1A SH2D2A SH2D3C SH3BP5 SH3PXD2B SH3RF3 SHANK2 SHANK3 SHB SHC3 SHMT2 SHROOM1 SIGLEC10 SIK1 SIL1 SIPA1 SIRPA SIRPG SIT1 SKAP1 SKIV2L SLA SLAMF1 SLAMF6 SLAMF7 SLAMF8 SLC15A2 SLC16A4 SLC17A8 SLC19A3 SLC1A4 SLC23A1 SLC2A6 SLC39A6 SLC3A2 SLC41A1 SLC43A3 SLC6A19 SLC6A6 SLC7A5 SLC7A7 SLC9A2 SLC9A7 SLCO1B3 SLCO3A1 SLCO5A1 SLFN11 SLFN12 SLFN5 SLIT2 SMAGP SMAP2 SMCHD1 SNAI2 SNAPC1 SNCAIP SND1 SNN SNX20 SORBS1 SORBS2 SORBS3 SORCS2 SORD SOX13 SOX17 SOX18 SP110 SP140 SPAG4 SPARC SPARCL1 SPATS2 SPCS1 SPCS3 SPG20 SPHK1 SPIB SPIRE1 SPOCK2 **SPP1** SPR SREBF1 SREBF2 SRGAP2 SRM SRPRB SRPX2 SSBP2 SSPN SSR1 SSR4 SSSCA1 SSTR1 ST3GAL1 ST3GAL5 ST6GALNAC4 ST8SIA1 ST8SIA4 STAP1 STARD10 STAT2 STAT4 STAT5B **STC1** STK10 STK17A STX2 STX7 STXBP1 SULF1 SULF2 SWAP70 SYK SYNC SYNJ2 SYT11 SYTL1 SYTL3 SYTL4 TACC2 TAGLN TBC1D10C TBCB TBCEL TBXAS1 TCF3 TCF4 TCFL5 TCL1A **TCN1** TDO2 TEK TEX30 TFAP2C TFEC TFPI TFPI2 TGFA TGFB1 TGFB1I1 TGFB2 TGFBI TGFBR1 TGFBR2 TGIF2 THBD **THBS2** THEMIS THEMIS2 THY1 TIE1 TIGIT **TIMP1** TIMP2 TIMP3 TKT TLDC2 TLN2 TLR10 TLR5 TLR6 TM4SF18 TM6SF1 TMC8 TMEM109 TMEM11 TMEM119 TMEM141 TMEM154 TMEM156 TMEM158 TMEM163 TMEM177 TMEM184B TMEM204 TMEM206 TMEM243 TMEM246 TMEM258 TMEM30B TMEM39A TMEM45A TMEM55A TMEM63B TMEM64 TMEM72 TMEM8B TMOD1 TMTC1 **TNC** TNF TNFAIP8 TNFRSF11B TNFRSF12A TNFRSF17 TNFRSF4 TNFRSF9 TNFSF13B TOM1L2 TOP2B TOR3A TP53I11 TP53INP1 TPM2 TPMT TPST1 TPST2 TRA2A TRABD2A TRAF1 TRAF3 TRAF3IP3 TRAM1 TRAM2 TRAT1 TRBC1 TRIB2 TRIM10 TRIM15 TRIM3 TRIM36 TRIM40 TRIM52 TRIM8 TRMT112 TRPA1 TRPS1 TRPV2 TSC22D1 TSC22D3 TSHR TSHZ2 TSPAN11 TSPAN2 TSPAN33 TSPAN4 TSPAN5 TSPAN9 TSPYL5 TSTA3 TTC22 TTC28 TTC39C TTC7B TTLL6 TTR TUBA1A TWIST1 TWSG1 TXK TXLNB TXNDC11 TXNDC15 TXNIP TYRP1 UBASH3A UBASH3B UBE2E2 UBE2J1 UCP2 UGCG UGGT1 UNC13B UPP1 UROD USP11 USP2 VAMP1 VASN VAV1 VCAM1 VCAN VEGFC VGLL3 VIM VIP VMO1 VOPP1 VPS13C VWF WAS WASF3 WDFY4 WDR19 WDR45 WDR45B WDR54 WFS1 WIPF1 WISP1 WNK2 WNK4 WNT2 WNT5A WWC1 WWC3 WWTR1 XBP1 XYLB YBX2 YPEL1 YPEL3 ZAK ZAP70 ZBTB25 ZBTB7A ZC3H6 ZCCHC11 ZEB1 ZFP36L1 ZFYVE9 ZHX2 ZNF101 ZNF117 ZNF134 ZNF175 ZNF211 ZNF219 ZNF256 ZNF260 ZNF275 ZNF281 ZNF292 ZNF302 ZNF329 ZNF347 ZNF358 ZNF383 ZNF420 ZNF423 ZNF432 ZNF439 ZNF443 ZNF493 ZNF521 ZNF532 ZNF552 ZNF606 ZNF615 ZNF618 ZNF708 ZNF75A ZNF75D ZNF768 ZNF83 ZNF831 ZNF85 ZNF862 ZNF91 ZSCAN18 ZZEF1 |
| Black module | 1398(42/150) | A1CF ABAT ABCA5 **ABCB1** ABCB10 **ABCG2** ABHD14B ABHD3 ABHD5 ABI1 ABRACL ACAA1 ACAA2 ACACB ACADM ACADS ACADSB ACAP2 ACAT1 ACBD5 ACOT13 ACOT8 ACOX1 ACOXL ACSF2 ACSL5 ACSM3 ACSS2 ACVR1B ACVR1C ACVR2A ADCY6 ADCY9 ADD3 ADGRA3 **ADH1C** ADH5 ADH6 ADIPOR2 AFG3L2 AFTPH AGAP1 AGGF1 AGL AGMAT AGPAT3 AGPS AIFM3 AIMP1 AK2 AK3 AKAP1 AKAP7 AKR1B10 AKTIP ALAD ALDH18A1 ALDH1B1 ALDH6A1 ALDH9A1 ALDOA AMN ANK3 ANKMY2 ANKRD13C ANKRD17 ANKRD27 ANKRD9 ANO10 ANO9 ANPEP AOC1 APLP2 **APOBEC3B** APOOL APPBP2 APPL1 APRT AQP11 **AQP8** AQR ARF3 ARHGAP12 ARHGAP18 ARHGAP21 ARHGAP42 ARHGAP44 ARHGAP5 ARHGEF10L ARHGEF15 ARHGEF9 ARL6IP1 ARL6IP6 ARMC8 ARRDC4 ARSD ARV1 ASAP2 ASAP3 ASB13 ASB7 ASCC3 ASH2L ATAD1 ATAD2B ATG4D ATG5 ATP23 ATP2B1 ATP5C1 ATP5G1 ATP5G3 ATP5L ATP5O ATP6V0D1 ATP6V1D ATP8A1 ATP8B1 ATP9A ATPAF1 ATXN7L3B AUH AVIL B3GALT5 B3GNT8 B4GALT4 BAD BAG5 BAIAP3 BCAP31 BCAR3 BCAS1 BCKDHB BCL2L15 BDH1 BDH2 BECN1 BEND3 BEST2 **BEST4** BICDL2 BLCAP BLOC1S1-RDH5 BMPR1A BPHL BPNT1 BTBD3 BTC BTNL3 BZW2 C10orf99 C11orf54 C14orf2 C15orf40 C15orf48 C19orf33 C1D C1orf106 C1orf115 C1orf131 C1orf21 C1orf210 C1orf53 C1QBP C1QTNF3-AMACR C21orf33 C2CD2L C2CD5 C2orf88 C4orf19 C5orf30 C5orf63 C6orf120 C6orf136 C6orf203 C7orf31 C8orf33 **CA1** CA2 CA4 **CA7** CAAP1 CABIN1 CACFD1 CALCOCO2 CAMK2D CAMK2G CAMK2N1 **CAPN13** CAPN2 CAPN9 CASD1 CASP6 CAST CCDC112 CCDC68 CCL7 CCNG1 CCNT2 CD58 CD6 CD70 CDC42BPA CDH17 CDH3 **CDHR1** CDHR5 CDKN2B CDR2L CDX1 CDX2 CEACAM4 CEACAM7 CEBPA CENPV CEP57 CEP70 CERCAM CERS6 CES2 CES3 CETN3 CFDP1 CFTR CGN CGRRF1 CHCHD10 CHD1 CHKA CHML CHMP2B CHMP4B CHMP4C CHN2 CHP1 **CHP2** CHPF CHPT1 CHST5 CIPC CISD1 **CKB** CLCA1 CLCN2 CLDN23  **CLDN8** CLIC5 CLIP2 CLMN CLN3 CLRN3 CLSTN1 CLSTN3 CLYBL CMAS CNGA1 CNIH4 CNKSR3 CNNM2 CNNM4 **CNTN3** COA5 COBL COG7 COPS5 COQ7 COQ9 COX11 COX4I1 COX5A COX5B COX6B1 COX6C COX7B COX7C CPEB3 CPM CPNE2 CPT1A CPT2 CRLS1 CRNKL1 CROT CRYL1 CS CST3 CTBP2 CTDSP2 CTDSPL CTH CTPS2 CTSA CUL3 CUL5 **CWH43** CXADR CXorf23 CXorf36 CYB5B CYC1 CYCS CYP2B6 CYP2B7P CYP2J2 CYP4F12 DAPK2 DBT DCAF11 DCUN1D4 DDAH2 DEFB1 DENND1B DENND4C DEPTOR DERA DHDDS **DHRS11** DHX32 DISP2 DLAT DLD DLEU1 DLG1 DNAJA3 DNAJC10 DNAJC19 DNM2 DNMBP DOCK1 DOCK5 DOLPP1 DOPEY1 DPY19L4 DSG2 DSP DST DTX4 DUS4L DUSP7 DYNC1LI2 DYRK2 DYRK3 EAPP ECH1 ECHDC1 ECI1 ECI2 EDA EDN3 EEA1 EFL1 EFNA1 EFNA4 EHHADH EIF4E3 EIF4EBP2 ELAC1 ELF3 ELF4 ELMO3 EMP1 ENDOD1 ENPP1 ENPP3 ENPP4 **ENTPD5** EPB41L3 EPB41L4B EPHX2 EPN3 EPS8 ERBB2 ERBB3 ERCC8 ERLIN2 ERMP1 ESD ESRP1 ESRRA ETFA ETFB ETFDH ETHE1 ETNK1 **EXPH5** EYA2 FABP1 FABP5 FAH FAHD1 FAM105A FAM110C FAM120AOS FAM126B FAM134B FAM13A FAM162A FAM171A1 FAM20B FAM213A FAM234A FAM47E-STBD1 FAM83B FAM8A1 FANK1 FAR1 FASTKD1 FASTKD2 FASTKD3 FBXL14 FBXL4 FBXO25 FBXO3 FBXO34 FCGBP FCGRT FCHO2 FDX1 FEM1C FES FGD4 FGFR3 FH FHDC1 FITM2 FKTN FLJ22763 FLNB FLT4 FLVCR1 FMO4 **FMO5** FMR1 FNBP1L FNIP2 FOXA1 FPGT FRAT2 FRK FRMD1 FRS2 FRYL FUT2 FXYD3 FZD5 GAB1 GAL GALNT1 GALNT12 GAS2L3 GBA3 GBAS GCFC2 GCLM GCNT1 GCNT2 GDA GDAP2 GDE1 GDPD2 GFPT1 GGH GGT6 GHITM GIN1 GIPC2 GLB1L2 GLCE GLOD4 GLOD5 GLTP GLYCTK GMCL1 GMFB GMIP GNA11 GNAQ GNE GNG12 GOLGA5 GOLPH3L GON7 GOT1 GPA33 GPBP1L1 GPD1L GPR160 GPRIN2 GRAMD1C GRAMD3 GRAMD4 GRHL2 GRSF1 GSTM4 GSTZ1 GTF2A2  **GUCA2A** **GUCA2B** GUCY2C GUF1 HADH HADHA HADHB HARS2 HCCS HDHD3 HEATR5A HECTD3 **HEPACAM2** HEPH HES5 HHLA2 HIBCH HIGD1A HINT1 HMGCL HMGCR **HMGCS2** HMGN2P46 HMGN3 HNMT HNRNPAB HNRNPH3 HOOK1 HOOK2 HOXA10 HOXA11-AS HOXA13 HOXA2 HOXA3 HOXA5 HOXA7 HOXB5 HOXB6 HOXB7 HOXB9 HPGD HSBP1L1 HSD11B2 HSD17B11 HSDL2 HUS1 IARS2 ID2 IDH3A IDH3B IFI27 IFT57 IFT74 IFT88 IGBP1 Igk IGSF9 IHH IL10RB IL21 ILDR1 ILVBL IMMP1L IMMT IMPA1 IMPA2 IMPACT INO80 INSC INSR IP6K2 IQGAP2 IREB2 IRF2BP2 ISCA1 ISCA2 ISX ITCH IVD IYD JUP KARS KBTBD11 KBTBD7 KCNK5 KCTD9 KDF1 KDM4A KHNYN KIAA0232 KIAA1107 KIAA1191 KIAA1211 KIAA1468 KIAA1522 KIAA1804 KIF13B KIFAP3 KITLG KLB KLC4 KLF2 KLF4 KLF5 KLHDC10 KLHL20 KLHL34 KLHL8 KLHL9 KLK1 KMT5B KRAS KRT12 KRT20 KTN1 **KYNU** LAIR2 LAMTOR4 LANCL3 LAPTM4B LCMT1 LCOR LDHD LGALS4 LGALSL LGR5 LIAS LIMA1 LIN7C LINC00483 LINC00526 LINC01133 LINC01268 LIPT1 LLGL1 LLGL2 LMBRD2 LMF2 LMTK2 LNX2 LOC100506730 LOC653602 LPAR5 LPCAT4 LPIN2 LRBA LRIG3 LRP10 LRP4 LRP6 LRPPRC LRRC1 LRRC19 LRRC25 LRRC31 LRRC57 LRRC75A LRRCC1 LRRFIP2 LTB4R LUZP1 LYPD8 LYPLA1 LYRM7 LZIC MAB21L3 MAGI1 MAGI3 **MAOA** MAP2K5 MAP2K6 MAPK11 MAPK3 MAPK6 MAPK7 MAPK9 MAPKAPK5 MARC2 MARCKS MARK2 MARVELD2 MARVELD3 MAST2 MAVS MB MBNL3 MBOAT1 MCCC1 MCCC2 MCM9 MDH1 ME2 MED4 MED7 MEG3 **MEP1A** MEP1B MEST METAP1 METTL10 METTL25 METTL2A METTL7B MFGE8 MFSD11 MFSD12 MFSD4A MFSD9 MGAT4A MGAT4B MGLL MICAL3 MICALCL MICU2 MID1 MIDN MIER1 MINPP1 MIR1204 MKKS MKRN1 MLLT3 MLXIP MMACHC MMP15 MMP28 MOB3B MOGAT2 MOGAT3 MPC2 MPND MPP7 MPST MRPL30 MRPL34 MRPL35 MRPL48 MRPS14 MRPS25 MRPS30 MRPS33 MRPS36 MRRF MS4A12 MS4A8 MTAP MTERF1 MTIF2 MTM1 MTMR11 MTMR2 MTMR4 MTX2 MUT MXI1 MYB MYO15B MYO1A MYO1D MYO9B **NAAA** NAB2 NAPEPLD NARS NAT1 NAT2 NBEAL1 NBR1 NCBP2-AS2 NCDN NCOA4 NCOR1 NCOR2 NDFIP2 NDUFA5 NDUFA6 NDUFAB1 NDUFB5 NDUFB8 NDUFS1 NDUFS2 NDUFS3 NECTIN3 NEDD4L NEK3 NEK7 NEO1 NET1 NFS1 NHSL1 NIPA2 NIPAL2 NIPAL3 NIPSNAP3A NIT1 NLN NMB NNT NOP16 NPTN NR1I2 NR3C2 NR4A3 NR5A2 NRARP NRIP1 NT5DC2 NTF3 NUAK2 NUBPL NUDT12 NUDT16P1 NUDT4P1 NUDT7 NVL NXPE1 NXPE4 OCEL1 OCLN OCRL OMA1 OPA1 OPLAH OPN3 ORC2 OTUD7B OVOL1 OVOL2 OXR1 OXSR1 P2RX4 P2RY1 **PADI2** PAK1 PALM PANK1 PANK3 PAQR3 PAQR8 PARD3 PARG PARM1 PATJ PBLD PBX2 PC PCBD2 **PCK1** PCK2 PDCD6IP PDE3A PDGFA PDGFB PDHA1 PDHB PDHX PDIK1L PDLIM1 PDLIM7 PDPK1 PDXP PDZD3 PECR PER2 PET117 PEX1 PEX11A PEX11B PEX19 PEX2 PEX26 PEX7 PFKFB2 PFKL PGAP1 PGAP2 PGAP3 PGGT1B PGM1 PGM3 PGPEP1 PGRMC1 PGRMC2 PHACTR4 PHF3 PHLPP1 **PHLPP2**  PHYH PHYKPL PID1 PIEZO1 PIGR PIGS PIGZ PINK1 PIP5K1B PIWIL4 PKIB PLA2G12B PLAGL2 PLCB4 PLCE1 PLD1 PLEKHA6 PLEKHB2 PLEKHG2 PLEKHG6 PLEKHH1 PLS1 PLSCR4 PLXNA2 PM20D2 PMM1 PMPCB PNKD PNPLA4 POLR2G POLR2H PPARA **PPARG** PPARGC1A PPFIA3 PPFIBP2 PPID PPIG PPIP5K1 PPIP5K2 PPM1A PPM1B PPP1CB PPP1CC PPP1R13B PPP1R14D PPP1R36 PPP2CB PPP2R3A PPP2R5D PPP2R5E PQLC1 PRAP1 PRDX6 PRELID2 PREPL PRKACB PRKAR2A PRKCI PRKCZ PRMT6 PROSC PRR13 PRR15 PRR15L PRR26 PRR5L PRRG1 PRRG2 PRSS8 PSD3 PTER **PTGDR** PTGES3 PTGR2 PTK6 PTK7 PTPN21 PTPRD PTPRF PTPRH PTPRK PTPRO PTPRR PTTG1IP PUM1 PUS10 PWWP2A PXMP2 PXMP4 PYROXD1 RAB11FIP1 RAB14 RAB17 RAB32 RABIF RALGAPA1 RAP1GAP RAPGEFL1 RASEF RASSF3 RASSF6 RAVER2 RBKS RCHY1 RDH13 REPS2 RETSAT RFK RGP1 RGS14 RHBDL2 RHOU RIDA RILP RIMS3 RIOK3 RMDN2 RMND1 RMND5A RNASEL RNF11 RNF125 RNF128 RNF138 RNF14 RNF5P1 RPE RPIA RPL15 RPL22 RPL31 RPP14 RPP30 RPS5 RPS6KA5 RPS6KA6 RRAS2 RRN3 RSBN1 RSPH3 **RUNDC3B** RUSC1 S1PR2 SACM1L SAMD12 SAMD13 SAMD9 SAP18 **SATB2** SATB2-AS1 SBF2 SCAF8 SCAP SCGB2A1 SCIN SCNN1B SCO2 SCP2 SCRN3 SDC3 SDC4 SDHC SDHD SEC14L2 SEC22B SEC23B SECTM1 SEH1L **SELENBP1** SEMA5A SEMA6A SEMA6D SEMA7A SENP6 SENP8 SEPHS2 SEPP1 SEPSECS SEPT4 SERBP1 SERF1B SERINC1 SERINC5 **SERPINB5** SERTAD4 SETD2 SETD9 SFXN4 **SGK2** SGMS1 SH3BGRL2 SH3RF1 SH3RF2 SHROOM3 SIAE SIAH1 SIK2 SIKE1 SIPA1L2 SIPA1L3 SKP1 SLC12A2 SLC13A2 SLC16A1 SLC16A5 SLC16A9 SLC17A4 SLC17A9 SLC20A2 SLC22A18AS SLC22A23 SLC22A5 SLC23A3 SLC25A12 SLC25A13 SLC25A20 SLC25A23 SLC25A25-AS1 SLC25A34 SLC25A4 SLC25A40 SLC25A43 SLC25A5 **SLC26A2** SLC26A3 SLC30A10 SLC35A1 SLC35A3 SLC35D1 SLC35D2 SLC35G1 SLC36A1 SLC37A4 SLC39A4 SLC39A5 SLC3A1 SLC44A1 SLC44A3 SLC51A SLC51B SLC9A3R1 SMAD4 SMAD7 SMAP1 SMC3 SMC5 SMIM14 SMIM19 SMIM20 SMIM8 SMNDC1 SMOX SMPDL3A SNRNP48 SNX24 SNX30 SNX4 SNX6 SNX7 **SOSTDC1** SOWAHA SPAG1 SPATA2 SPECC1L SPHAR SPHK2 SPIN1 SPINK1 SPINT1 SPINT2 SPIRE2 SPOPL SPPL2A SPPL2B SPPL3 SPRYD7 SPTLC3 SRI SRSF8 SS18L1 SSBP3 SSH3 ST13 ST14 ST3GAL2 STAG1 STAM2 STAP2 STARD7 STK3 STK38 STK38L STK39 STRADB STRN STX12 STX19 STXBP3 STYK1 SUCLA2 SUCLG2 SUDS3 SULT1A1 SULT1A2 SULT1B1 SUMF1 SUN1 SUOX SUPV3L1 SVIP SYNJ2BP-COX16 SYTL2 TADA2A TAOK3 TATDN1 TBC1D12 TBCE TC2N TCEA3 TCEB1 TCF7L2 TCTA TDP2 TDRD3 TDRD7 TEN1-CDK3 TESC TEX11 TEX264 TFCP2L1 TGFB3 TGOLN2 THAP9 THG1L THNSL1 THRB THSD4 TIAL1 TINAG TIPRL TJP3 TLE2 TLK1 TLK2 TLR3 TM2D1 TM4SF5 TMBIM6 TMC4 TMCC3 TMCO1 TMEM120A TMEM123 TMEM127 TMEM128 TMEM129 TMEM132A TMEM133 TMEM144 TMEM14B TMEM14C TMEM168 TMEM170A TMEM171 TMEM184C TMEM186 TMEM192 TMEM209 TMEM251 TMEM37 TMEM38B TMEM41B TMEM45B TMEM80 TMEM87B TMEM98 TMEM9B TMLHE TMPRSS4 TMTC4 TNIK TNMD TOM1L1 TOMM20 TOMM40L TOX3 TP53INP2 TP53TG1 TPD52 TPM1 TPRG1L TPRN TPSG1 TRADD TRAF3IP2 TRAF4 TRAK1 TRAK2 TRANK1 TRHDE TRIM2 TRIM29 TRPM4 **TRPM6** TRPM7 TSHZ1 TSN TSPAN12 TSPAN3 TSPAN6 **TSPAN7** TSPAN8 TST TSTD1 TTC13 TTC19 TTC30A TTC30B TTC9 TUBAL3 TUFT1 TXNDC9 TXNL4A UAP1 UBAC1 UBE2A UBE2D2 UBE4A UBL3 UBTD1 UBXN2B UGDH UGP2 UGT1A1 UGT1A3 UGT2A3 UGT8 ULK3 UNG UQCC1 UQCR11 UQCRB UQCRC1 UQCRC2 UQCRFS1 USH1C USMG5 USP25 USP30 USP54 USP8 VAPA VAV3 VDAC3 VDR VIL1 VILL **VIPR1** VPS13D VPS26A VPS36 VPS37B VPS41 VPS4B VSIG10 VSIG2 VTI1B VWA1 VWA8 WASL WDR20 WDR34 WDR78 WIPF2 WSB2 WWP1 XK XRCC4 ZADH2 ZBTB10 ZBTB7C ZC3H14 ZCCHC14 ZDHHC23 ZDHHC3 ZFP62 ZG16 ZKSCAN1 ZNF124 ZNF140 ZNF148 ZNF165 ZNF195 ZNF217 ZNF326 ZNF33B ZNF462 ZNF468 ZNF518A ZNF564 ZNF57 ZNF575 ZNF577 ZNF675 ZNF678 ZNF704 ZNF789 ZNF823 ZNHIT3 ZNRF2 ZNRF3 ZSWIM5 ZYX ZZZ3 |
| Green module | 282(0/150) | ACLY ADK ADRA2C ANKRD39 ANP32E ARHGAP11A ASF1B ASPM AURKA AURKB BARD1 BCL2L12 BCS1L BET1 BIK BIRC5 BLMH BRCA2 BUB1 BUB1B BYSL CBX1 CCNA2 CCNB1 CCNB2 CCNE2 CCNF CCT7 CDC20 CDC25A CDC25C CDC45 CDC6 CDC7 CDCA2 CDCA3 CDCA5 CDCA7L CDCA8 CDK1 CDK2 CDK4 CDKN3 CENPE CENPK CENPM CENPN CENPW CEP55 CEP78 CETN2 CFAP36 CHEK1 CKAP2L CKS1B CKS2 CLPP CMSS1 COPS3 COQ2 COX20 CTPS1 DDB1 DDIAS DDX18 DDX19A DDX39A DEPDC1B DHCR7 DIAPH3 DLGAP5 DNA2 DNMT1 DONSON DPAGT1 DTL DTYMK ECT2 EIF3I EIF4A3 ELAC2 ERH ESCO2 ESPL1 EXO1 EXOSC8 EZH2 FANCG FANCI FBXO5 FDPS FEN1 FOXM1 GEMIN4 GINS4 GLMN GNL2 GPI GPSM2 GSTO1 GTSE1 H2AFX H2AFZ HAPLN1 HELLS HJURP HMGA1 HMGB2 HMGN2 HMMR HPRT1 HSD17B10 HSD17B4 IARS IDI1 ILF2 IMP4 INSIG1 IQGAP3 KIAA0101 KIAA1524 KIF11 KIF14 KIF18A KIF18B KIF20A KIF23 KIF2C KNL1 KNSTRN KPNA2 LARS2 LDHB LMNB1 LMNB2 LYAR MCM10 MCM2 MCM3 MCM5 MCM6 MCM7 MELK MKI67 MLH1 MND1 MORC4 MRPL17 MRPL9 MSH6 MTHFD2 MVD MYBL2 MYDGF NCAPD2 NCAPG NCL NDC80 NEK2 NHP2 NOLC1 NOP2 NPM1 NPM3 NUBP1 NUDT1 NUP133 NUP93 NUSAP1 ODC1 ODF2 OIP5 OXCT1 PARK7 PARP2 PBK PCNA PCSK9 PFAS PFN2 PHF19 PLK1 PLK4 PLP2 PNPLA7 POLD2 PRC1 PRMT1 PRPS1 PRR11 PSMB3 PSMB4 PSMC3IP PSMC4 PSMC5 PSMD14 PSME4 PTTG1 RACGAP1 RAD23A RAD51AP1 RAD51C RAD54L RAN RANBP1 RANGAP1 RFC2 RFC4 RNASEH2A RPL26L1 RPL39L RRM1 RRM2 RRS1 RUVBL1 RUVBL2 SAP30 SASS6 SCAMP3 SCD SEC13 SETD7 SHCBP1 SKA1 SLC35B1 SNRPA SNRPA1 SNRPB SNRPB2 SNRPC SNRPD2 SNRPD3 SNRPF SNX17 SOD1 SPAG5 SQLE SRP72 SSR2 SSRP1 STIP1 STRA13 TACC3 TAF5 TALDO1 TCF19 TIMELESS TK1 TM6SF2 TMEM97 TOP2A TPP2 TPX2 TRAP1 TRIM28 TRIM59 TRIP13 TRMU TROAP TTK TUBA4A TUBB TUBG1 TYMS UBAP2L UBE2C UBE2S UBE2T UBFD1 UFD1L UHRF1 USP14 VARS VCP VPS72 VRK1 WBSCR22 WDHD1 WDR46 WEE1 XPOT YIF1A |
| Cyan module | 209(0/150) | AAK1 ABCD3 ACTB ADAM10 ADAM9 AHCYL2 ANKS4B AP1AR APPL2 ARF6 ARFGEF1 ARFGEF2 ARFIP1 ARSA ASAH1 ASCC1 ASF1A ATE1 ATF1 ATF5 ATL2 ATOH1 ATP1B3 AZIN1 BCL2L13 BSG CAB39 CARM1 CASK CAT CCDC6 CCNDBP1 CD164 CD46 CDC40 CDC42SE2 CDH1 CDV3 CEP63 CGGBP1 CHMP1B CITED2 CLIC1 CMTM4 CNOT1 CPNE3 CRY2 CTDNEP1 DAZAP2 DCTD DDOST DIAPH2 DNAJC3 DPP7 DSC2 DSCR3 EFCAB14 EGLN1 EHF ELF1 EML4 ENO1 EPB41L2 EPRS ERI1 EZR F2RL1 FAF2 FAM175B FAM76A FBXO28 FBXW11 FEM1B FOXJ3 GALC GAPDH GDPD5 GLE1 GLUD2 GNA13 GOLGA7 GPD2 GSR H1F0 HNF4G HSP90B1 IBTK IDE IFNGR1 IKZF5 INPP5A IPO13 IRF6 ITGA6 KBTBD2 KIAA1033 KIF5B KLF3 LPAR2 MAN1A2 MAP4 MAP7 MAPK14 MAX MBNL1 MCL1 MED15 MED20 MET MIER3 NCBP1 NCKAP1 NCOA2 NFE2L2 NSMAF NXT2 OAT P4HB PACS2 PACSIN2 PAFAH1B1 PAK2 PAPOLA PAQR5 PDE8A PDIA6 PDP1 PIK3R1 PJA2 PKP2 PLEC PLEKHF2 POF1B POLR2A POLR3C PPP2R1A PSEN1 PTGER4 PTP4A1 R3HCC1L RAPGEF3 RB1 RBL2 RBM47 RELL2 RHBDD3 RND3 RNF114 RNF6 ROCK2 RRN3P1 SCAMP1 SEC31B SEC61A1 SEPT10 SEPT11 SERPINB1 SH3D19 SHOC2 SLC39A14 SLC4A4 SNRNP70 SNW1 SNX13 SNX2 SOCS6 SORT1 SOS2 SPAG9 SPRED2 SRPRA STAG2 STX3 SURF4 SV2A TAB2 TCIRG1 TECPR1 THRA TJP1 TM9SF4 TMED2 TMEM30A TMX1 TOX4 TRIB1 TRIM23 TSPAN18 TTC37 TXLNG UBE3B UBE4B UBR1 USO1 USP10 USP12 VRK2 YTHDF3 ZBTB6 ZFP41 ZNF22 ZNF24 ZNF253 ZNF273 ZNF324 ZNF41 ZNF43 ZNF655 ZNF780A |
| Grey60 module | 157(15/150) | ACSL1 ADAM8 ADGRE2 ADGRG3 AIF1 ALOX5AP ALPL **AQP9** ATP11A **BCL2A1** BCL6 BEST1 C3AR1 C5AR1 CASS4 CCL4 CCR1 CD300A CD33 CDC42EP2 CEBPB CFP CLEC4A CLEC5A CMTM2 CSF2 CSF2RB CSF3 **CSF3R** CXCL5 **CXCL8** CXCR1 CXCR2 CYP27B1 DSE DYSF EHD1 EMILIN2 F5 FCAR FCER1G FCGR1CP FCGR2A FCGR3B FCN1 FFAR2 FGR FPR1 FPR2 FRZB FST **G0S2** **GBP5** GCA GK GLT1D1 GNLY GPR65 GZMB HCAR3 HCK HK3 HSPA6 ICAM1 IFNG IGSF6 IL10 IL11 IL17RA IL18RAP IL1A **IL1B** IL1RAP **IL1RN** IRAK2 JUNB KCNJ15 KCNJ2 KRT23 LCP2 LIF LILRA1 LILRA2 LILRA3 LILRA5 LILRB1 LILRB2 LILRB3 LST1 LYN MAP3K8 MGAM MMP25 **MNDA** MUCL1 MX2 MYO1F NABP1 NAMPT **NCF2** NDP NFE2 NFKBIA NINJ1 NLRC4 OLR1 OSM P2RY13 PDE4B PFKFB3 PHC2 PILRA PIM1 **PLEK** PLK3 PRF1 PROK2 PTAFR PTGS2 PTPRE RAB40B RGS18 RGS2 RHOG RNASE2 RNF144B **S100A12** **S100A8** **S100A9** SAMSN1 SIRPB1 SLC11A1 SLC22A4 SLC25A37 SLC2A3 SNX10 **SOCS3** **SRGN** STEAP4 STX11 SUCNR1 TAGAP TLR1 TLR2 TLR4 TLR8 TMEM71 TNFAIP2 TNFAIP3 TNFAIP6 TNFRSF1B TREM1 TYROBP **VNN2** XPO6 ZFP36 ZNF267 |
| Salmon module | 123(11/150) | ACKR4 ADAR APOL1 ASPHD2 ASS1 B2M BATF2 **BIRC3** BTN3A1 BTN3A2 BTN3A3 C19orf66 C2 C5orf15 CARD16 CASP1 CASP8 CBR3 CD274 CD74 **CDC25B** CFLAR CIITA CXCL10 **CXCL11** DESI1 DHX58 DMXL2 DNAJA1 DTX3L EPSTI1 ETV7 FBXO6 GBP1 GBP2 GBP4 GRAMD2 GRIN3A HCP5 HLA-DMA HLA-DMB HLA-DPA1 HLA-DPB1 HLA-DRA HLA-DRB1 HLA-F HLA-G HPS5 HSD3B7 **IDO1** IFI35 IFI44 IFI44L IFI6 IFIH1 IFIT2 IFIT3 IFIT5 IFITM1 **IFITM2** **IFITM3** IL15 IL15RA IRF1 IRF7 IRF9 ISG15 ISG20 JAK2 KCNJ10 LAP3 LPGAT1 MCUB MDK MLKL MX1 N4BP1 NFE2L3 NMI OAS2 OASL PARP14 PARP9 PLEKHS1 PML POMT1 PPA1 PSMB10 PSMB8 PSMB9 PSME1 PSTPIP2 RAB24 **RARRES3** RIPK2 RNF19B RNF213 RNF24 RSAD2 RTP4 SAMD9L SHISA5 SOCS1 SP100 STAT1 STAT5A **STOM** TAP1 TAP2 TGM2 TMSB10 TRIM21 **TRIM22**  TRIM69 TYMP **UBD** UBE2L6 USP18 VAMP5 VSNL1 **WARS** XAF1 ZBP1 |
| midnightblue module | 97(18/150) | ADGRG6 **ADM** AGPAT4 ANGPTL4 **ANXA1** ANXA2 ANXA2P2 ANXA3 ARFGAP3 ASRGL1 ATP13A2 AZGP1 BACE2 BAIAP2L1 C2CD4A **C4BPA** **C4BPB**  **CASP5** CD55 CEACAM6 **CFB** CLDN1 **CXCL1** **CXCL2** CXCL3 DEFB4B **DMBT1** DPY19L1 **DUOX2** **DUOXA2** ERO1A ERRFI1 FAM167B FAM83A FHL2 GALNT2 GLRX GPRC5A HIF1A ITGA2 KCND3 KDELR3 LAMC2 **LCN2** LDHA LIPG **LPCAT1** LRG1 MRAP2 NFKBIZ **NOS2** **OLFM4** PDIA4 PDK2 **PDZK1IP1** PF4 PFKP **PI3** PIM3 PLAUR PLIN3 PLOD2 PPP2R5B RHOD RND1 RTEL1 RTEL1-TNFRSF6B S100A11 SAA2 SAA2-SAA4 SERPINB3 SERPINB4 SHH SLC2A1 SLC5A1 **SLC6A14** SLC6A20 SLC7A11 SLCO4A1 SLPI SNPH SPNS2 SRD5A3 STAT3 STRIP2 STS TM4SF1 TMC5 TMEM92 **TNIP3** TPK1 TRIM47 UNC5CL **VNN1** VSIG1 XDH ZG16B |
