# Supplementary material for: Identification of genes and functional coexpression modules closely related to ulcerative colitis by gene datasets analysis
Source: PeerJ. 2019 Nov 13;7:e8061. doi: 10.7717/peerj.8061 (PMC6858811; doi:10.7717/peerj.8061)
Supplement: Supplemental Information 3 [file peerj-07-8061-s003.doc]

**Table S3 Compared to other 4 RNA micro-arrays studies of UC**

| **author & year** | **sample size of UC patients** | **Sample type** | **Significant differential expression genes** |
| --- | --- | --- | --- |
| Our research.2019 | 328 | [tissue](../../../../C:/Users/dazhu/AppData/Local/youdao/dict/Application/8.5.3.0/resultui/html/index.html#/javascript:;) [biopsy](../../../../C:/Users/dazhu/AppData/Local/youdao/dict/Application/8.5.3.0/resultui/html/index.html#/javascript:;) | MMP1 REG1A DMBT1 DUOX2 REG3A DUOXA2 LCN2 MMP10 MMP3 SELL S100A9 S100A8 CXCL13 CXCL8 PI3 CHI3L1 REG1B CXCL11 MMP7 OLFM4 PIM2 SLC6A14 CFB IL1B MMP12 SPINK4 MMP9 STC1 IL1RN VNN1 CCL19 IDO1 CCL20 CXCL10 TIMP1 CD27 PLAU CSF3R TNIP3 TFF1 ADM C2 S100P IGFBP5 LAX1 CFI CXCL1 AQP9 SERPINB5 WARS DEFA5 KYNU PECAM1 CTSK NOS2 THBS2 SRGN CCR7 CCL11 GNA15 PLEK SELP RARRES3 NCF2 GBP5 MNDA CXCL6 SERPINA3 ANXA1 C4BPB ANGPTL2 CCL18 CEMIP SELE SOCS3 C4BPA TRIM22 CXCR4 BCL2A1 IFITM2 CXCL2 PDZK1IP1 REG4 IFITM3 TNC TCN1 CASP5 LPCAT1 STOM BGN UBD CDC25B OSMR GEM S100A12 ALDOB SPP1 BIRC3 G0S2 VNN2 AQP8 CLDN8 ABCG2 HMGCS2 PCK1 GUCA2B GUCA2A PHLPP2 FMO5 RUNDC3B SLC26A2 HAVCR1 CHP2 EXPH5 MEP1A TSPAN7 SELENBP1 MT1F HSD17B2 CDHR1 CKB ENTPD5 ADH1C MT1H VIPR1 CA1 CAPN13 ABCB1 SATB2 TMIGD1 PPARG CWH43 MAOA CA7 APOBEC3B NAAA SGK2 PTGDR BMP3 BEST4 SOSTDC1 CD177 MT1G PADI2 CNTN3 HEPACAM2 TRPM6 DHRS11 CNTFR MT1M |
| FengWu, et al.2007 | 5 | [tissue](../../../../C:/Users/dazhu/AppData/Local/youdao/dict/Application/8.5.3.0/resultui/html/index.html#/javascript:;) [biopsy](../../../../C:/Users/dazhu/AppData/Local/youdao/dict/Application/8.5.3.0/resultui/html/index.html#/javascript:;) | CORO1A MMP12 PECAM1 TLN1 TIMP1 IFI30 POU2AF1 CLU TNFRSF7 PTGDS CD79A DEFA5 UBD CCL11 IGFBP5 ECGF1 FSCN1 ATM NOTCH3 PI3 NIP210 ARID5A PDK3 CTSH LCP1 STOM SEMA6A PHLPPL MRPS6 SC5DL SCP2 UGDH CAST ADAMDEC1 DNAH9  EFNA1 FGFR3 MUT PCK1 GGH ASAHL ACADM UGT2B28 ENTPD5 ENPP4 MTMR11 ACOX1 NEDD4L TSPAN7 PTPRR VPS13A PLOD2 DYRK2 GUCA2A GUCA2B SRI EDN3 PRDX6 SELENBP1 AKAP9 PIK3R1 F2RL1 LGALS2 CHD1 HNF4G MLLT2 MYB NR3C2 SATB2 ZNF217 CCNT2 KLF5 ATP2B1 EXPH5 SLC16A1 SCAMP1 TNPO1 SLC26A2 AQP8 COBL FAM8A1 FLJ13910 FRMD4B HIST1H4C LOC63928 LOC92482 RSBN1 |
| Noble, et al.2008 | 67 | [tissue](../../../../C:/Users/dazhu/AppData/Local/youdao/dict/Application/8.5.3.0/resultui/html/index.html#/javascript:;) [biopsy](../../../../C:/Users/dazhu/AppData/Local/youdao/dict/Application/8.5.3.0/resultui/html/index.html#/javascript:;) | SAA1 DEFA5 DEFA6 S100A8 S100A9 MMP3 MMP7 IL8 TLR4 TNIP3 CCL20 ABCB1 |
| TEPPEI KOBAYASHI, et al.2012 | 6 | Peripheral whole blood cells | LGALS3 IGHG3 MRP14 HBG2 IL-1R2 |
| Núria Planell, et al.2012 | 15 | [tissue](../../../../C:/Users/dazhu/AppData/Local/youdao/dict/Application/8.5.3.0/resultui/html/index.html#/javascript:;) [biopsy](../../../../C:/Users/dazhu/AppData/Local/youdao/dict/Application/8.5.3.0/resultui/html/index.html#/javascript:;) | COL1A2 CXCL1 CXCL3 IL8 NFKBIZ REG1A VCAN WNT5A AQP8 CHI3L1 LILRB2 MMP1 MMP3 TGFBI TIMP1 ABCG2 ACOX1 ACSL4 ADH6 AQP3 CXCL5 DEFB1 GJA1 IL1B IL1RN IL6R ME1 MMP10 REG4 RUNDC3B S100P SERPINB5 SLC16A1 SMAD7 TFF1 |
